# Analysis of *GPR126* polymorphisms and their relationship with scoliosis in Marfan syndrome and Marfan-like syndrome in Mexican patients

**DOI:** 10.17305/bb.2023.9268

**Published:** 2023-12-01

**Authors:** Maria Elena Soto, Giovanny Fuentevilla-Alvarez, Solange Gabriela Koretzky, Gilberto Vargas-Alarcón, Yazmín Estela Torres-Paz, Sergio Enrique Meza-Toledo, Israel Pérez-Torres, Claudia Huesca-Gómez, Ricardo Gamboa

**Affiliations:** 1 Department of Immunology, Instituto Nacional de Cardiología Ignacio Chávez, México City, México; 2 Cardiovascular Line Department in American British Cowdray (ABC) Medical Center, México City, México; 3 Department of Physiology, Instituto Nacional de Cardiología Ignacio Chávez, México City, México; 4 Department of Biochemistry, Escuela Nacional de Ciencias Biológicas, Instituto Politécnico Nacional (IPN), México City, México; 5 Editorial Department, Instituto Nacional de Cardiología Ignacio Chávez, México City, México; 6 Research Direction, Instituto Nacional de Cardiología Ignacio Chávez, México City, México; 7 Department of Cardiovascular Biomedicine, Instituto Nacional de Cardiología Ignacio Chávez, México City, México

**Keywords:** G protein-coupled receptor 126 (*GPR126*), Marfan syndrome (MFS), single nucleotide polymorphism (SNP), scoliosis

## Abstract

Marfan syndrome (MFS) is an inherited connective tissue disorder. As the spinal growth depends on the delicate balance of forces, conditions that affect musculoskeletal matrix often lead to spinal deformities. A large cross-sectional study revealed a 63% prevalence of scoliosis among patients with MFS. Multi-ethnic genome-wide association studies and analyses of human genetic mutations showed that variations and mutations of G protein-coupled receptor 126 (*GPR126*) locus are associated with multiple skeletal defects, including shorter stature and adolescent idiopathic scoliosis. The study included 54 patients with MFS and 196 control patients. The DNA was extracted from peripheral blood using the saline expulsion method and single nucleotide polymorphism (SNP) determination was carried out using TaqMan probes. Allelic discrimination was performed by RT-qPCR. Significant differences in genotype frequencies were found for SNP rs6570507 in relation to MFS and sex (recessive model, odds ratio [OR] 2.46, 95% confidence interval [CI] 1.03–5.87; *P* ═ 0.03) and rs7755109 (overdominant model, OR 0.39, 95% CI 0.16–0.91; *P* ═ 0.03). The most significant association was found in SNP rs7755109, where the frequency of genotype AG was significantly different between MFS patients with scoliosis and those without (OR 5.68, 95% CI 1.09–29.48; *P* ═ 0.04). This study, for the first time, examined the genetic association of SNP *GPR126* with the risk of scoliosis in patients with connective tissue diseases. The study revealed that SNP rs7755109 is associated with the presence of scoliosis in Mexican patients with MFS.

## Introduction

Marfan syndrome (MFS) is an inherited connective tissue disorder caused by heterozygous mutations in the *FBN1* gene, which encodes the extracellular matrix protein fibrillin-1 [1]. MFS is considered as one of the most lethal conditions in Mendelian inheritance [2]. The *FBN1* gene can undergo a range of mutations, such as missense or nonsense mutations, insertions or deletions that occur within or outside of the frame, and mutations affecting exonic or splice sites. In approximately 40% of MFS cases, mutations in the *FBN1* gene lead to haploinsufficiency. Meanwhile, dominant negative mutations account for approximately 60% of cases. These mutations modify the components of fibrillin-1 and thus affect interaction with other proteins from the extracellular matrix. A variety of effects of these mutations have been described at the protein level; they include changes to the secondary structures of proteins, which can lead to a delay in protein secretion or an increased susceptibility. Furthermore, truncation codon mutations are linked with the onset of severe skeletal and skin lesions [3]. As the spinal growth depends on the delicate balance of forces, conditions that affect musculoskeletal matrix often lead to spinal deformities. A large cross-sectional study revealed a 63% prevalence of scoliosis among patients with MFS. However, many of these curves were minor and only 10%–20% needed treatment. The curve patterns resembled those seen in patients with idiopathic scoliosis, with thoracolumbar and thoracic curves being the most common types. Interestingly, there was a higher prevalence of triple curves noted in these cases [4, 5]. Adolescent idiopathic scoliosis (AIS) is a complex three-dimensional deformity of the spine, including variable degrees of changes across the frontal, sagittal, and axial planes. It affects 2%–3% of the adolescent population [6] with the incidence of 1 in every 300–1000 live births; women are affected more frequently than men [7]. While both genetic and epigenetic factors have been associated with AIS, the specific molecular and cellular mechanisms that underlie these axial skeletal disorders still have not been elucidated.

Numerous loci have been identified as potential candidate genes for AIS, including G protein-coupled receptor 126 (*GPR126)*, *CHL1*, *LBX1*, and *GAL3ST4* [8]. The *GPR126*, a member of the adhesion family, is involved in various developmental defects. Multi-ethnic genome-wide association studies and analyses of human genetic mutations have demonstrated that variations and mutations of *GPR126* locus are linked to several skeletal defects, including shorter stature, AIS [9], congenital arthrogryposis multiplex [10], and periodontitis [11]. Given this background, the objective of this study was to determine whether the single nucleotide polymorphisms (SNPs) rs6570507 and rs7755109 are associated with thoracic deformity in patients with MFS.

## Materials and methods

### Patient population

This was a combined retrospective and prospective study. It included 54 subjects diagnosed with MFS or a similar condition who were evaluated from 1998 to 2018. Their clinical files were reviewed and assessed by a trained rheumatologist to ensure compliance with the Ghent criteria. Specific characteristics of each patient were identified through imaging techniques, which confirmed a Haller index greater than 3.2. Furthermore, the study included the detection and investigation of scoliosis and dural ectasia.

One hundred ninety-six patients who did not have MFS or idiopathic scoliosis were included as the controls. These patients underwent similar evaluation processes, involving clinical history review and direct evaluation at the time of sample collection in order to exclude connective tissue diseases and scoliosis. A simple radiography of the lumbosacral spine was used as the imaging method.

### Echocardiogram and magnetic resonance

An echocardiography cardiologist performed a transthoracic echocardiogram to evaluate the presence of mitral, aortic, and tricuspid valve prolapse in the apical 4-chamber plane. The Haller index was determined using measurements obtained from a magnetic resonance imaging scan. This index was calculated by dividing the maximum transverse diameter of the thorax by the minimum anteroposterior diameter, with measurements made from the anterior edge of the vertebral body to the posterior border of the sternum [12].

### Blood samples

Five mL of venous blood was collected from each participant in tubes containing ethylenediaminetetraacetic acid (EDTA), as well as in tubes without an anticoagulant, after 12 h of fasting. The blood was then centrifuged to separate the plasma, which was used for the determination of the lipid profile.

### DNA extraction

White blood cells were isolated from whole blood through the lysis of erythrocytes using SLR 1Xsolution. Following this, the leukocyte packet was incubated with 10% SDS and K proteinase (10 mg/mL) at 37 ^∘^C overnight to allow for enzymatic digestion. DNA was then extracted using the saline expulsion method. Subsequently, the DNA concentration was measured using a spectrophotometer (BioPhotometer plus) at a wavelength of 260/280 nm.

### Determination of polymorphisms

The SNPs were selected based on previously published reports (Table 1). Frequencies reported in other populations were reviewed using HapMap, including SNPs with minor allele frequencies greater than 1%. Various polymorphisms were identified using TaqMan probes with the CFX96TM Touch Real-Time PCR Detection System. Real-time PCR is a reliable method for the rapid detection of single nucleotide changes. The probes were synthesized by the Applied Biosystems Company. Six µL of TaqManTM Universal PCR Master Mix was used in a reaction volume of 10 µL at a final concentration of 10 ng/µL of DNA, 700 nM of primers, and 100 nM of the fluorophore-labeled probe. The reaction conditions were set to 10 min at 95 ^∘^C followed by 40 cycles of 15 s at 92 ^∘^C and 1 min at 60 ^∘^C. The fluorescence levels of the PCR products were quantified using the CFX MasterTM Software, Bio-Rad.

**Table 1:** Information on the SNPs studied

**Gene SNP**	***GPR126* rs6570507**	***GPR126* rs7755109**
Chromosome	6	6
SNP type	Intron Variant	Intron Variant
MAF	0.31	0.38

Data obtained from the 1000 Genomes Project. SNPs: Single nucleotide polymorphisms; MAF: Minor allele frequency.

### Ethical statement

Informed consent was obtained from each participant after detailed explanation of the purpose and procedures involved in the research study. This was done in accordance with the stipulations of the Declaration of Helsinki, as modified at the Tokyo Congress, Japan [13]. The research was approved by the Ethical, Biosecurity, and Investigation Committees of the Instituto Nacional de Cardiologia Ignacio Chavez (Registration number 18-1084).

### Statistical analysis

Data were analyzed using the SPSS version 19 (SPSS Inc., Chicago, USA). The risk factors were dichotomized in the form of presence or absence of clinical criteria. A descriptive analysis was conducted for all variables, with results expressed as mean ± standard deviation. The comparison between groups was made using the Student’s *t*-test for continuous variables. For variables that did not have a normal distribution, the Mann–Whitney *U* test or the Kruskal–Wallis test was used.

The models evaluated in SNPs were the following: dominant (11 vs 12 + 22), overdominant (12 vs 11+ 22), recessive (22 vs 12+11), codominant 1 (11 vs 12), codominant 2 (11 vs 22), additive (2(22) + 12 vs + 11), and allelic (1 vs 2). The Hardy–Weinberg equilibrium (HWE) for controls and patients was determined using a chi-square test. Polymorphism analysis was calculated using SPSS version 18 (SPSS Chicago, IL, USA) and EPISTAT statistical program (Version 5.0; USD Incorporated 1990, Stone Mountain, Georgia). *P* values were obtained based on the number of comparisons performed. A *P* value of less than 0.05 was considered statistically significant. The relative risk was calculated as an odds ratio (OR) with 95% confidence intervals (CI).

## Results

### Clinical characteristics

A total of 289 Mexican subjects were studied at the Ignacio Chávez National Institute of Cardiology. All participants met the inclusion criteria. The inclusion criteria specified patients with MFS who met more than two Ghent classification criteria, and all selected controls had no comorbidities. The study population consisted of 99 subjects with a mean age of 23 ± 13, while the control group comprised 190 patients with a mean age of 26 ± 11. Out of 99 patients with connective tissue disease included, 54 were with MFS and 45 with Marfan-like disorders. The characteristics of the study population are summarized in Table 2. This SNP study analyzed all individuals from both the study and the control group.

**Table 2:** Frequencies and percentages of the type of disease and demographic characteristics by sex

**Variable**	**Total, *n* (%)**	**Men, *n* (%)**	**Women, *n* (%)**	***P***
	99 (100)	45 (45)	54 (55)	
Age (years)	23 ± 13	21 ± 10	25 ± 14	NS
*Ghent Criteria*				
Family history	45 (45)	17 (38)	28 (52)	NS
Lens luxation	33 (33)	12 (27)	21 (32)	NS
Aortic dilation	66 (67)	28 (64)	38 (70)	NS
Systemic score >7	73 (74)	34 (76)	39 (72)	NS
*Types of Connective Tissue Disease*		
MFS	54 (55)	23 (52)	31 (57)	NS
LDS	19 (19)	9 (20)	10 (19)	NS
NCTD	11 (11)	6 (13)	5 (9)	NS
MASS syndrome	3 (3)	2 (4)	1 (2)	NS
EDS	5 (5)	2 (4)	3 (6)	NS
BHS	4 (4)	3 (7)	1 (2)	NS
Stickler syndrome	1 (1)	0	1 (1)	NS
*Thorax Deformity*				
Scoliosis	68 (68)	31 (69)	37 (69)	NS
Pectus excavatum	25 (25)	18 (40)	7 (13)	0.001
Pectus carinatum	51 (52)	21 (47)	30 (56)	NS

NS: No significance; MFS: Marfan syndrome; LDS: Loeys-Dietz syndrome; NCTD: Non-connective tissue disease; MASS: Mitral valve prolapse, Aortic root diameter at upper limits of normal for body size, Stretch marks of the skin, and Skeletal conditions similar to Marfan síndrome; EDS: Ehlers Danlos syndrome; BHS: Beals–Hecht syndrome.

### Haplotypes analysis

Haplotype analysis was performed using Haploview software. However, no significant associations were found between the studied polymorphisms when comparing the group with disease with the control group. The same analysis was also conducted in patients with thoracic deformities, however, no significant differences were found in any allelic combinations.

### Genotype and allelic frequencies of *GPR126* (rs6570507, rs7755109)

We evaluated the allelic and genotypic frequencies of the two SNPs to determine if there is an association between the mutation and the presence of thoracic deformities. When comparing the study group with the control group, we found no association or significant differences in any of the evaluated models (data not shown). Subsequently, we carried out a sex-based stratification of both study groups. We evaluated different models, and the results are presented in Table 3.

**Table 3:** Allelic and genotypic frequency of SNPs rs6570507 and rs7755109 in controls and patients with Marfan syndrome in relation to sex

**SNP**	**Genotype frequency,** ***n* (%)**			**Model**	***P*1 value** **OR (95% CI)**	***P*2 value** **OR (95% CI)**
rs6570507	11	12	22			
Control			
Men	11 (9)	57 (49)	53 (46)	Dominant	0.26 0.47 (0.12–2.27)	0.70 1.77 (0.33–9.3)
Women	6 (8)	27 (36)	42 (56)			
				Over dominant	0.63 1.38 (0.55–3.4)	**0.05** **0.40 (0.17–0.95)**
MFS			
Men	4 (15.9)	9 (47.7)	10 (36.4)	Recessive	0.22 1.38 (0.55–3.4)	**0.03** **2.46 (1.03–5.87)**
Women	2 (9.6)	18 (50)	11 (40.4)	Codominant 1	0.24 0.43 (0.11–1.66)	0.69 2 1 (0.36–11.03)
				Codominant 2	0.45 1.92 (0.51–7.27)	1 1.27 (0.22–7.19)
				Additive	0.26 0.48 (0.14–1.64)	1 1.08 (0.20–5.57)
				Allelic	0.69 0.82 (0.42–1.59)	0.22 0.63 (0.33–1.20)
rs7755109	11	12	22			
Control			
Men	60 (52)	45 (39)	10 (8)	Dominant	0.49 1.41 (0.57–3.49)	0.09 2.22 (0.94–5.22)
Women	45 (60)	27 (36)	5 (6)			
				Over dominant	0.81 1.20 (0.47–3.07)	**0.03** **0.39 (0.16–0.91)**
MFS			
Men	10 (43)	8 (22)	5 (18.1)	Recessive	0.13 0.34 (0.10–1.120)	0.67 2.08 (0.23–18.5)
Women	12 (39)	18 (51)	1 (3)	Codominant 1	1 0.93 (0.34–2.56)	**0.04** **0.4 (0.16–0.95)**
				Codominant 2	0.12 0.33 (0.09–1.18)	1 1.33 (0.14–12.51)
				Additive	0.67 1.24 (0.52–2.94)	0.14 2.02 (0.87–4.68)
				Allelic	0.15 1.69 (0.87–3.28)	0.23 1.50 (0.78–2.87)

Bold indicates statistical significance; 11: homozygous dominant; 12: heterozygous; 22: homozygous recessive; OR: Odds ratio; *P*1: Control Men vs MFS Men; *P*2: Control Women vs MFS Women; CI: Confidence interval; SNP: Single nucleotide polymorphism; MFS: Marfan syndrome.

In the case of the SNP rs6570507, after stratification by sex and comparing groups, we found that in the SNP rs6570507 when comparing the GG genotype in women with MFS (40.4%) and control women (56%) there is a significant difference (OR 2.46, 95% CI 1.03–5.87; *P* ═ 0.03).

On the other hand, when reviewing the results obtained from the SNP rs7755109, we found significant differences when comparing the genotype frequencies of the AG genotype in healthy women and women with MFS (51%) (OR 0.39, 95% CI 0.16–0.91; *P* ═ 0.03). In the same way, we observed significant differences in the GG genotype (OR 2.46, 95% CI 1.03–5.87; *P* ═ 0.03).

Based on the above results, the next step was to perform a comparison of genotypic and allelic frequencies, stratifying the MFS population by type of thoracic deformity, including scoliosis, pectus excavatum, pectus carinatum, and dural ectasia.

When reviewing the results of the SNP rs6570507, no association was found with any thoracic deformity and the genotypes present (Table 4). However, the results of SNP rs7755109 showed significant differences when comparing AG genotype frequencies in patients with MFS with scoliosis and without scoliosis (OR 5.68, 95% CI 1.09–29.48; *P* ═ 0.04) (Table 5).

**Table 4:** Allele and genotypic frequency of the rs6570507 A/G polymorphism in patients with Marfan syndrome with and without thoracic deformity

**SNP**	**Genotype frequency,** **n (%)**			**Model**	***P*1 value** **OR (95% CI)**	***P*2 value** **OR (95% CI)**	***P*3 value** **OR (95% CI)**	***P*4 value** **OR (95% CI)**
rs6570507	11	12	22					
**Scoliosis**			
Positive	4 (9)	24 (55)	15 (34)	Dominant	0.59 0.46 (0.05–5.94)	0.34 2.69 (0.31–22.32)	0.66 (0.46 (0.03–3.16)	1 0.77 (0.09–9.5)
Negative	2 (18)	3 (27)	6 (54)					
				Over dominant	0.17 3.36 (0.78–14.46)	1 1 (0.31–3.21)	1 0.86 (0.29–2.5)	1 0.88 (0.28–2.7)
**Pectus excavatum**	
Positive	3 (19)	8 (50)	5 (31)	Recessive	0.39 0.44 (0.11–1.70)	0.65 0.62 (0.18–2.15)	0.45 1.8 (0.58–5.55)	1 0.85 (0.26–2.76)
Negative	3 (7)	19 (50)	16 (43)					
**Pectus carinatum**			
Positive	2 (7)	13 (48)	12 (45)	Codominant 1	0.21 0.25 (0.03–1.99)	0.37 2.37 (0.39–14.38)	0.66 0.53 (0.08–3.45)	1 0.7 (0.10–4.69)
Negative	4 (15)	14 (51)	9 (34)	Codominant 2	1 0.8 (0.11–5.58)	0.31 3.2 (0.48–21.16)	0.38 0.37 (0.05–2.51)	1 0.87 (0.12–5.94)
**Dural ectasia**								
Positive	4 (10)	20 (50)	16 (40)	Additive	0.61 1.8 (0.30–10.79)	0.34 0.35 (0.06–1.90)	0.41 2.31 (0.39–13.46)	1 1.23 (0.21–7.27)
Negative	2 (13)	7 (43)	7 (41)					
				Allelic	0.82 1.26 (0.46–3.44)	0.39 1.58 (0.68–3.69)	0.42 0.66 (0.30–1.47)	1 1.02 (0.43–2.43)

11: Homozygous dominant; 12: Heterozygous; 22: Homozygous recessive; OR: Odds ratio; *P*1: Scoliosis vs non-scoliosis; *P*2: Pectus excavatum vs non-pectus excavatum; *P*3: Pectus carinatum vs non-pectus carinatum; *P*4: Dural ectasia vs non-dural ectasia; CI: Confidence interval; SNP: Single nucleotide polymorphism.

**Table 5:** Allelic and genotypic frequency of the rs7755109 A/G polymorphism in patients with Marfan syndrome with and without thoracic deformity

**SNP**	**Genotype frequency,** ***n* (%)**			**Model**	***P*1 value** **OR (95% CI)**	***P*2 value** **OR (95% CI)**	***P*3 value** **OR (95% CI)**	***P*4 value** **OR (95% CI)**
rs7755109	11	12	22					
**Scoliosis**			
Positive	15 (35)	24 (56)	4 (9)	Dominant	0.09 0.30 (0.05–1.46)	0.53 0.56 (0.16–1.93)	0.78 1.36 (0.45–4.04)	0.89 1.33 (0.37–4.69 )
Negative	7 (64)	2 (18)	2 (18)					
				Over dominant	**0.04** **5.68 (1.09–29.48)**	1 1.11 (0.34–3.57)	0.78 0.74 (0.25–2.16)	1 0.90 (0.26–3.05)
**Pectus excavatum**							
Positive	5 (30)	8 (50)	3 (20)	Recessive	0.59 0.46 (0.08–2.92)	0.34 2.69 (0.48–15.07)	1 1 (0.18–5.45)	1 0.66 (0.10–4.10)
Negative	17 (45)	18 (47)	3 (8)					
**Pectus** **carinatum**			
Positive	12 (44)	12 (44)	3 (11)	Codominant 1	**0.007** **0.18 (0.03–0.98)**	0.76 0.66 (0.18–2.43)	0.77 1.40 (0.45–4.38)	1 1.25 (0.33–4.69)
Negative	10 (37)	14 (52)	3 (11)	Codominant 2	1 1.07 (0.16–7.31)	0.42 0.29 (0.05–1.94)	1 1.20 (0.20–7.31)	1 1.70 (0.24–12.17)
**Dural** **ectasia**								
Positive	17 (43)	19 (48)	4 (10)	Additive	0.19 2.49 (0.71–8.70)	0.39 1.98 (0.60–6.55)	0.78 0.75 (0.26–2.15)	0.82 0.72 (0.21–2.44)
Negative	5 (36)	7 (50)	2 (14)					
				Allelic	0.45 0.6 (0.22–1.78)	0.32 0.59 (0.25–1.38)	0.84 1.17 (0.53–2.59)	0.76 1.27 (0.52–3.08)

Bold indicates statistical significance. 11: Homozygous dominant; 12: Heterozygous. 22: Homozygous recessive. OR: Odds ratio; *P*1: Scoliosis vs non-scoliosis; *P*2: Pectus excavatum vs non-pectus excavatum; *P*3: Pectus carinatum vs non-pectus carinatum; *P*4: Dural ectasia vs non-dural ectasia; CI: Confidence interval; SNP: Single nucleotide polymorphism.

## Discussion

In diseases that damage connective tissue, significant abnormal changes often occur in the anatomical structure of bone and cartilage, particularly in the thoracic region of the skeleton [14]. Apart from changes in the spinal structure, these diseases can result in thoracic asymmetry and conditions like pectus excavatum or carinatum parallel with cardiovascular damage [15]. These structural bone changes frequently occur in correlation to aortic dilatation [16] or other types of cardiovascular damage [17–20], which are serious conditions that can reduce life prognosis and necessitate early surgical intervention. These interventions can become more complicated when managing structural bone deformities in the context of reparative aortic surgery [21].

In this study, we evaluated two polymorphisms of the *GPR126* gene (rs6570507 and rs7755109) related to idiopathic scoliosis, since there are no studies that have focused on syndromic conditions in connective tissue disorders.

Upon reviewing the results for the first evaluated SNP rs6570507, we found that the GG genotype occurred less frequently in female patients with MFS compared with female control patients. This could suggest that the absence of the GG genotype may be a potential risk factor. Limited information is available regarding the relationship between this SNP (rs6570507) and scoliosis in women. However, a study by Kou et al. [22], which utilized Genome-Wide Association Study (GWAS) methods on the *GPR126* gene and idiopathic scoliosis, demonstrated that this SNP was the most significantly associated with scoliosis in a Japanese population. Similarly, a study conducted on a Han Chinese population found a significant association between SNP rs6570507 and idiopathic scoliosis. Further supporting these findings, zebrafish model studies have identified *gpr126* in the locus, as a promising candidate gene for idiopathic scoliosis susceptibility, given the fact that these studies on zebrafish have already demonstrated *gpr126*’s role in both growth and ossification of the developing spine and in neurological development [22]. Our study is pioneering in investigating scoliosis associated with syndromic conditions. However, when reviewing the results for SNP rs6570507, we did not find statistically significant associations between thoracic deformities and the presence of scoliosis in patients with MFS. This lack of significant findings could be due to the relatively small sample size. Nevertheless, our results showed a higher frequency of the AG genotype in subjects with MFS. The observed OR was 3.36 (95% CI 0.78–14.46, *P* ═ 0.17), and although this difference did not reach statistical significance, it does suggest a rising trend of the AG genotype in subjects with scoliosis. When reviewing the results for the SNP rs7755109, we observed a higher frequency of the AA genotype in female patients with MFS. This suggests that the presence of the AA genotype could potentially act as a risk factor for MFS.

Until now there are no studies that showed changes in relation to sex, but this observed difference could be related to the musculoskeletal condition. Although several studies have explored the genetic association with overall height, the specific genetic basis distinguishing the skeletal components contributing to height still remains unidentified. A comprehensive report involving nearly 20,000 individuals from the United Kingdom and the Netherlands provided statistically significant evidence that 17 genomic regions are associated with sex, including 4 gene regions associated with the size of skeletal structures like the spine, femur, and hip axis length [23, 24]. This could potentially account for why we found differences in genotype frequency in both SNPs among women in our study, while no such differences were observed in men.

In the case of SNP rs7755109, the results showed that the AG genotype had a higher prevalence in patients with scoliosis, which could indicate a risk factor. This is consistent with a study conducted in a Chinese population, in which they provided evidence of a strong association of intronic SNPs of the *GPR126* gene with susceptibility to AISrs6570507 A > G and rs7774095 A > C [25, 26]. One reason why the *GPR126* gene might be related to scoliosis is its involvement in chondrogenic differentiation. Studies have shown that during the chondrogenic differentiation process of human mesenchymal stem cells (hMSCs), there is a high level of *GPR126* mRNA expression. Additionally, recently discovered SNPs have been found to be associated [27] with cartilage development, potentially offering valuable insights into the etiology and pathogenesis of AIS. These intriguing findings suggest the need for further studies on tissue samples from patients with either idiopathic or syndromic scoliosis [28].

It has been reported that intronic SNPs affect transcription factor binding sites and native splice sites [29]. The 3’ untranslated region (3’UTR) is postulated to serve as a control area with the capacity to influence the localization, translation, and stability of mRNA. This potential regulatory activity could affect the interaction with regulatory molecules like microRNAs (miRNAs) [30]. Therefore, more studies need to be carried out on intronic regions that contain regulatory elements, such as enhancer/attenuator elements that regulate transcription. Despite these findings, we still need to answer critical questions about the precise spinal elements and signaling pathways crucial for maintaining proper spinal alignment. Further, we need to understand how potential treatments can reduce clinical damage in patients. These studies together provide evidence that biomechanical defects in cartilage and connective tissues may be due to defective *GPR126* signaling and could underlie the pathogenesis of AIS. It also suggests that stimulation of cAMP in spinal tissues may offer a promising treatment approach in order to interrupt the onset, progression, and severity of scoliosis. In relation to our findings on the gene polymorphisms compared with other studies, our results support earlier studies about the association of these polymorphisms with the syndrome although ethnicity can have an impact on the findings. Although we acknowledge that our main limitation is the sample size, there is still a greater tendency of finding the AG genotype in the rs6570507 A/G polymorphism in patients with scoliosis and the AA genotype of the same polymorphism with pectus excavatum. Despite that the frequency trends need to be confirmed with larger sample sizes for statistical significance, these results guide performing mRNA and protein expression studies.

In experimental animal models, it has been found that during development and adulthood, cells that express the *GPR126* (Adgrg6) play an important role in a variety of tissues and organs, such as the heart, sciatic nerve, cartilage, and ear. These cells are identified as cells exposed to mechanical stimuli, suggesting a potential role for *Gpr126* in mechanical signal transduction. This insight lays the groundwork for future research into this possibility [31].

For the first time, we have investigated the genetic association between the *GPR126* SNP and the risk of scoliosis in patients with connective tissue diseases. We discovered an association between the rs7755109 SNP and the prevalence of scoliosis among Mexican patients with MF. The results closely mirror those seen in patients with idiopathic scoliosis, which includes subjects whose diagnostic determinations in the context of connective tissue disorders may not be fully precise. This finding encourages a more comprehensive genetic study to assess the role of various mutations in these patients, who often present with multi-organ damage and syndromic clinical manifestations.

### Limitations

Magnetic resonance (MR) evaluation was not performed in the control group. It should be noted that connective tissue diseases are rare conditions with a low incidence and prevalence, even at a national reference center. Hence, while the sample size may not be large, it is noteworthy considering the number of individuals affected by these conditions. Nevertheless, a limitation of our study is that we did not undertake a multicenter investigation involving other countries.

## Conclusion

Our work is one of the first evidences in the Mexican population that the SNP rs7755109 is associated with syndromic scoliosis. This finding suggests that genetic regulation of the structural elements within the axial skeleton and connective tissue plays a vital role in maintaining the alignment of the vertebral column and other bone structures.
